# Overexpression of *PvFAD3* Gene from *Plukenetia volubilis* Promotes the Biosynthesis of α-Linolenic Acid in Transgenic Tobacco Seeds

**DOI:** 10.3390/genes13030450

**Published:** 2022-02-28

**Authors:** Guo Liu, Zhihua Wu, Xiuhua Shang, Yan Peng, Liqiong Gao

**Affiliations:** Research Institute of Fast-Growing Trees, Chinese Academy of Forestry, 30 Mid Renmin Avenue, Zhanjiang 524022, China; cercwuzhua2889@caf.ac.cn (Z.W.); cercshxh@caf.ac.cn (X.S.); cercpengy@caf.ac.cn (Y.P.); riftgaolq@caf.ac.cn (L.G.)

**Keywords:** *Plukenetia volubilis*, *PvFAD3*, expression pattern, subcellular localization, overexpression

## Abstract

The ω-3 fatty acid desaturase (*FAD3*) gene encodes a rate-limiting enzyme in the synthesis of α-linolenic acid. In this study, homologous cloning was used to obtain the full-length sequence of the *PvFAD3* gene of *Plukenetia volubilis*. The full-length DNA sequence was 1871 bp long, with 8 exons and 7 introns. The structural analysis of the amino acid sequence revealed that the PvFAD3 protein contained three histidine-conserved regions and an endoplasmic reticulum retention signal. The real-time reverse transcription-polymerase chain reaction performed for determining the expression patterns of the *PvFAD3* gene in different tissues of *P. volubilis* showed that *PvFAD3* expression was highly expressed in the fast oil accumulation stage of seed. The analysis of subcellular localization assay in epidermal cells of tobacco (*Nicotiana benthamiana*) leaves showed that the PvFAD3 protein was mainly localized in the endoplasmic reticulum. Seed-specific overexpression vectors were constructed, and *Agrobacterium*-mediated genetic transformation was performed to obtain transgenic tobacco plants overexpressing *PvFAD3*. The results of fatty acid assays performed using harvested seeds showed a significant increase in α-linolenic acid content, a dramatic decrease in linoleic acid content, and an obvious increase in oil content in transgenic tobacco seeds. Collectively, the *PvFAD3* gene of *P. volubilis* was confirmed as a key enzyme gene for α-linolenic acid synthesis; thus, indicating that the *PvFAD3* gene can be used for fatty acid fraction improvement in oilseed plants.

## 1. Introduction

*Plukenetia volubilis L.* is a perennial woody vine of the Euphorbiaceae family, which can produce fruits in the same year of planting and within 2–3 years after it enters the productive period of up to 10 years, with great economic value [1]. With an oil content of 35% to 60% [2], *P. volubilis* seeds are free from any toxins and harmful substances and contain more than 90% unsaturated fatty acids. This makes them one of the vegetable oils with the highest unsaturated fatty acid content worldwide, with a ratio of 1:0.75 for ω-3:ω-6 unsaturated fatty acids, which is closest to the absorption ratio of the human body as one of the best edible oils [3,4]. *P. volubilis* seeds contain 35.60% of α-linolenic acid, next to flaxseed and perilla seed in acid content, and are one of the main sources of α-linolenic acid product development [5,6].

α-Linolenic acid (C18:3, ALA) is an essential, plant-based, ω-3 polyunsaturated fatty acid that is obtained through the diet. It is a precursor for synthesizing two essential factors (EPA, C20:5, and DHA, C22:6) in the human body [7,8]. The synthesis and accumulation of α-linolenic acid are closely related to the FAD3 gene, and the synthesis of α-linolenic acid in plant seeds is catalyzed by the FAD3 protein for linoleic acid in the endoplasmic reticulum [9]. Several studies have reported that FAD3 overexpression can significantly increase α-linolenic acid content in leaves, roots, fruits, seeds, and other tissues of *Arabidopsis thaliana* [10], *Brassica napus* [11], and *Lycopersicon esculentum* [12]. The FAD3 gene has been successfully cloned and expressed in various plants, including *Glycine max* [13], *B.* *napus* [11], *Jatropha curcas* [14], *Arachis hypogaea* [15], *Erilla frutescens* [16], *Paeonia suffruticosa* [17], and *Camellia sinensis* [18]. In most plants, the FAD3 gene is highly expressed in seeds, and the expression is closely related to α-linolenic acid synthesis in seeds. For instance, the *JcFAD3* gene was overexpressed in *A. thaliana* seeds, in which the α-linolenic acid level was significantly increased by 20.50% to 24.94% compared to wild-type seeds [14]. Specific expression of the *FAD3c* gene of *G. max* in sesame seeds resulted in a 4.91% increase in the specificity of α-linolenic acid levels in sesame seeds [19]. The vector with *EuFAD3–1* overexpression increased the α-linolenic acid content of transgenic tobacco seeds by 4.27% after transfer into tobacco [20]. Our research team analyzed the transcriptomic data of *P. volubilis* seeds at various growth stages obtained using second-generation high-throughput sequencing technology and discovered that the *PvFAD3* gene in *P. volubilis* seeds was overexpressed during the rapid period of oil accumulation. [21]. We presumed that the extremely high expression level of PvFAD3 was closely related to the efficient accumulation of α-linolenic acid, which is consistent with the findings of Wang et al. [22,23] on *P. volubilis* seeds.

In this study, we aimed to characterize the expression patterns further. Biological functions of the *PvFAD3* gene in *P. volubilis* were selected to clone the full-length coding sequence of the *PvFAD3* gene, and the sequence structure was analyzed by bioinformatics analyses. Real-time reverse transcription-polymerase chain reaction (qRT-PCR) was performed to assess the expression of the gene in different tissues of *P. volubilis.* The expression of the *PvFAD3* gene was also characterized by subcellular localization. Seed-specific overexpression vectors were constructed, and *Agrobacterium*-mediated genetic transformation was performed to obtain transgenic tobacco plants overexpressing the *PvFAD3* gene. This study provides a basis for further understanding of the synthesis and metabolic mechanism of α-linolenic acid in oil plants and lays a theoretical foundation for improving the seed oil composition to get high yield and high quality oil plants.

## 2. Materials and Methods

### 2.1. Materials

Three-year-old *P. volubilis* plants were grown in the South China Experimental Nursery, located in Zhanjiang City in the southwest of Guangdong Province, China. Fresh leaves were used as the material for extracting genomic DNA and RNA. *Nicotiana benthamiana* (2*n* = 38) was used for the subcellular localization and plant transformation as the test material.

### 2.2. Cloning of the DNA Sequence of the PvFAD3 Gene 

The genomic DNA of *P. volubilis* was extracted using the modified CTAB method, and the integrity of the genomic DNA was examined using 1% agarose gel electrophoresis. Full-length cDNA and genomic sequences of *PvFAD3* were amplified from *P. volubilis* with FAD3-F and FAD3-R (Supplementary Table S1) as primers. The primers were designed based on the transcript sequence of the PvFAD3 (Unigene0043398) in *P. volubilis* seed transcriptome (SRR8205220). The PCR reaction system included 1.5 µL of the forward primer, 1.5 µL of the reverse primer, 25 µL of 2× Ex Taq buffer, 1 µL of dNTP (10 mmol·L^−1^), 5 µL of cDNA, 1 µL of Ex Taq enzyme (5 U/µL, TaKaRa Bio, Beijing, China), and 15 µL of ddH_2_O. The reaction procedure was as follows: predenaturation at 94 °C for 2 min, denaturation at 94 °C for 30 s, annealing at 53 °C for 30 s, extension at 72 °C for 1 min, for 35 cycles; hold at 72 °C for 10 min, store at 4 °C. The PCR products were subjected to agarose gel electrophoresis, recovered, ligated into the pMD19-T vector (Takara, Dalian, China), and sequenced by transforming the positive clones obtained from *Escherichia coli*.

### 2.3. Bioinformatics Analysis

Splign, a spliced-alignment tool in NCBI, was used to analyze the structure of PvFAD3. Theoretical isoelectric point (pI) and molecular weight of PvFAD3 were predicted by ExPASy (http://web.expasy.org/compute_pi/, accessed on 12 November 2021). Multiple sequence alignment was performed using Bioedit 7.0. A phylogenetic tree of PvFAD3 and other ω-3 fatty acid dehydrogenase was constructed by PhyML 3.0 software (maximum likelihood method), and bootstrap analysis using 1000 replicates. The analysis of transmembrane protein structure was carried out using TMHHMM Server V2.0 (http://www.cbs.dtu.dk/services/TMHMM/, accessed on 9 November 2021). The secondary structure and tertiary structure models of submits of PvFAD3 protein were predicted using Phyre v2.0 (http://www.sbg.bio.ic.ac.uk/phyre2/, accessed on 18 November 2021). 

### 2.4. Expression Pattern of PvFAD3 Gene

Primer 6.0 was used to design the qRT-PCR primers (Unigene0043398-F and Unigene0043398-R) (Supplementary Table S1), and total RNA was extracted from the samples using an RNAprep pure plant kit (TIANGEN, Beijing, China) and reverse transcribed using a HiScript II Q RT SuperMix for qPCR (+gDNA wiper) (Vazyme, Nanjing, China). The RNA quality for all samples was assessed by NanoDrop 2000 spectrophotometer (Thermo Fisher, Waltham, MA, USA). The qRT-PCR amplifications were performed with a Thermo Scientific PikoReal 96 Real-Time PCR System using iQ SYBR Green Supermix (TaKaRa Bio, Beijing, China) according to the manufacturer’s instructions. PCR product specificities were confirmed by melt curve analysis. Triplicate samples of qRT-PCR for PvFAD3 were prepared in a total volume of 20 µL that contained 4 µL cDNA template, 10 µL 2× ChamQ SYBR qPCR Master Mix (Vazyme, Nanjing, China) and 0.4 µL in forward and reverse primers. The actin gene (Unigene0042747) was used as an internal reference standard. The PCR reaction procedure was as follows: predenaturation at 95 °C for 1.5 min, denaturation at 95 °C for 5 s, annealing at 60 °C for 15 s, extension at 72 °C for 20 s, for 40 cycles; hold at 72 °C for 10 min, store at 4 °C. Statistical analyses were performed using the 2^−ΔΔCt^ method [24] (Livak & Schmittgen 2001) to calculate the relative expression of the *PvFAD3* gene in the root, stem, leaf (Mature leaf and Young leaf), pericarp, and seeds (the initial stage, Pv-1, and the fast oil accumulation stage, Pv-2) of *P. volubilis*, and multiple comparisons were performed using the Q-test, and significant differences were analyzed by a range test (significant differences at *p* < 0.05).

### 2.5. Construction of Fusion Vector and Overexpression Vector

The RNA prep pure plant kit (TIANGEN, Beijing, China) was used to extract total RNA, and then it was reverse transcribed into cDNA using the PrimeScript™ RT reagent kit (TaKaRa Bio, Beijing, China). The *PvFAD3* gene transcript sequence was used to create primers to construct a subcellular localization vector (YFP-FAD3-F and YFP-FAD3-R, Supplementary Table S1), and the cDNA was used as a template for PCR. The amplification product was cloned by homologous recombination into the EcoRI and SpeI sites of the vector p131-YFP to generate the expression vector of the fusion protein p131-PvFAD3-YFP.

The primers of the overexpression vector were designed on the basis of the sequence of Unigene0043398 (1301-FAD3-F and 1301-FAD3-R, Supplementary Table S1). Using the p131-PvFAD3-YFP plasmid as a template, the ORF (Open reading frames) sequence of the *PvFAD3* gene was amplified, and the PCR amplification product was cloned into BglII and BstEII of the expression vector pCAMBIA1301 by homologous recombination to replace the GUS (-β-Glucuronidase) gene in the vector. The target fragment was recovered using 1% agarose gel electrophoresis and ligated to a large fragment of the pCAMBIA1301 vector T4-DNA Ligase and incubated overnight at 4 °C. The ligated product was transferred to *E. coli* Top10 receptor cells using heat-shock transformation and coated on LB plates containing kanamycin (Kan) and incubated overnight at 37 °C. Positive clones were screened, and recombinant plasmids were extracted and identified using enzymatic digestion. The vector pCAMBIA1301-PvFAD3 was created. 

### 2.6. Subcellular Localization

The constructed subcellular localization vector plasmid was transformed with *Agrobacterium tumefaciens* GV3101, and p131–35S-YFP empty vector used as a control was set up. After incubating overnight, *Agrobacterium* was harvested by centrifugation and suspended in a suspension solution composed of 10 mM MgCl_2_, 10 mM MES, and 100 µM acetosyringone (AS). It was then mixed with *A. tumefaciens* containing an ER-mCherry subcellular marker plasmid [25], and then infiltrated into tobacco leaves at the 4-to 5-leaf stage after incubation for 3 h at room temperature. The colonies were identified by PCR without error and subjected to *A. tumefaciens* injection of *N. benthamiana* leaves. The expression and distribution of fluorescent proteins in epidermal cells of tobacco leaves were observed 3–5 days after the infiltration using a Leica TCS SP8 (Leica Microsystems GmbH., Wetzlar, Germany)) at an excitation wavelength of 514 nm (YFP) and 587 nm (mCherry).

### 2.7. Generation of Transgenic Tobacco and the Determination of Fatty Acid Composition

The overexpression vector pCAMBIA1301-PvFAD3 was used to obtain transgenic tobacco by leaf disc-mediated genetic transformation in *A. tumefaciens*. A pair of specific primers was designed on the basis of the pCAMBIA1301-PvFAD3 vector sequence (35S-F and FAD3-R2(562), Supplementary Table S1), and PCR was performed by using the leaf genomic DNA of wild type and transgenic tobacco as templates. The PCR results were verified by 1.0% agarose gel electrophoresis, and transgenic plants were obtained. Gas chromatography was used to determine the fatty acid composition of mature seeds from transgenic plants and wild tobacco (Agilent 7890A, Palo Alto, CA, USA). The specific operation method used was the same as that used by Liu et al. [26].

## 3. Results

### 3.1. Cloning and Sequence Analysis of the PvFAD3

**Figure 1:**
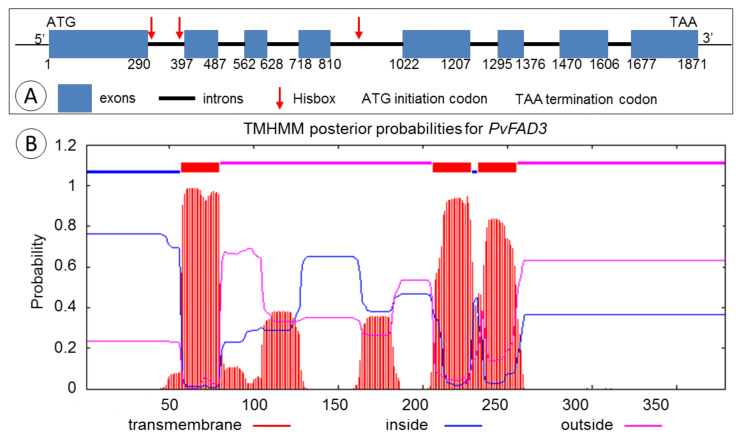
(**A**) Genomic structure of *PvFAD3* gene. The blue bars represent exons. Thick black lines represent introns, red arrows represent hisbox, ATG indicates initiation codon, and TAA indicates termination codon; (**B**) The transmembrane helix antenna analysis of PvFAD3. The red lines indicate transmembrane, blue lines indicate inside and pink lines indicate outside.

The genomic DNA and full-length cDNA sequence of *PvFAD3* were successfully cloned from *P. volubilis* on the basis of the Unigene0043398 transcript sequence. The sequence analysis showed that PvFAD3 had a full length of 1871 bp ORF (Figure 1A). Comparison of the PvFAD3 cDNA sequence with the transcript sequence of Unigene0043398 indicated that the *PvFAD3* gene contained eight exons, and the genomic DNA fragment was interrupted by seven introns at the ‘GT’ and ‘AG’ sites. The length of eight exons ranged from 67 to 290 bp with an average length of 142 bp, whereas the length of the seven introns ranged from 71 to 212 bp with an average length of 105 bp. In the introns, the (A+T) content was 65.53%, and the (G+C) content was 34.47, which is consistent with the higher AT content than GC content in the intron region. NCBI Blast search yielded the protein ID number QGR25673.1, and the sequence of PvFAD3 had high similarity to the other 34 plants ω-3 fatty acid dehydrogenases, ranging from 73.58% to 87.07% (Supplementary Table S2), with the highest similarity (87.07%) to the ω-3 fatty acid dehydrogenase of *Ricinus communis*, which is also a member of the Euphorbiaceae family.

PvFAD3 had a 1140 bp ORF encoding 379 amino acids with a molecular weight of 43.53 kD, as deduced by the sequence analysis. The predicted protein’s theoretical isoelectric point was calculated to be 7.79, and the stability coefficient was 34.07, indicating that the protein was stable. The hydrophilicity value of the protein was –0.102, indicating that it was hydrophobic. The protein with the aliphatic amino acid coefficient of 92.80 was aliphatic. According to the prediction of the transmembrane domain of PvFAD3 protein, three transmembrane domains were found (Figure 1B), and the corresponding transmembrane positions were I56–79O205–228I232–255O (“O” means outside the membrane and “I” means inside the membrane). Further analysis revealed that PvFAD3 did not have a signal peptide splicing site at the N-terminal, indicating that the PvFAD3 protein probably was located in the endoplasmic reticulum, and the protein may not be secreted to other sites across the membrane.

**Figure 2:**
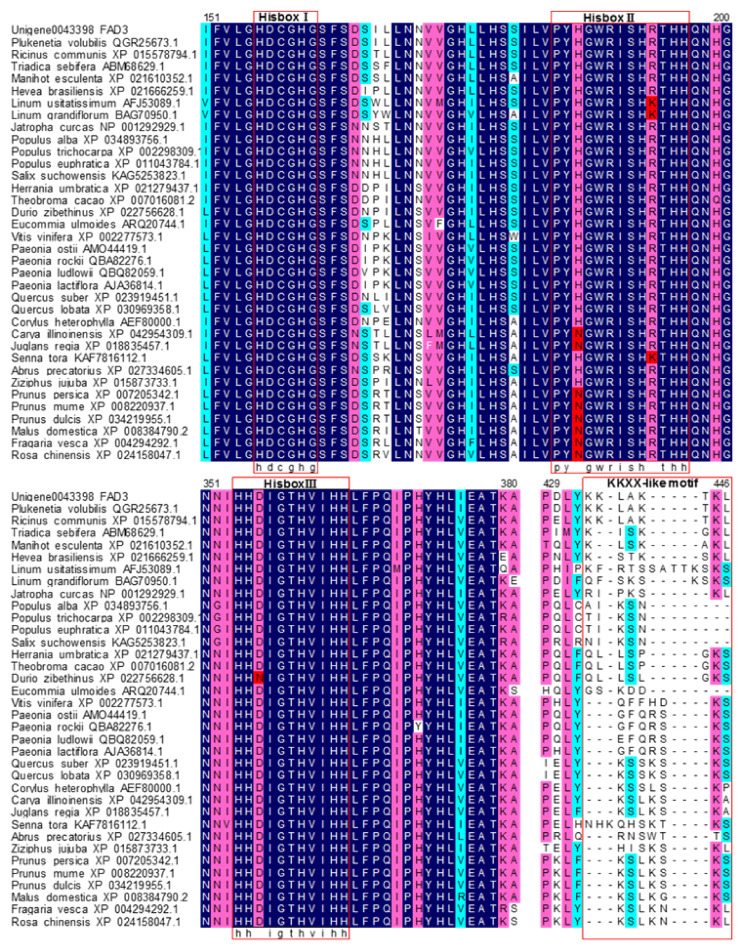
The alignment result of FAD3 amino acid sequences. The red boxes indicate Hisbox I, Hisbox II, Hisbox III, and KKXX-like motif, respectively.

The amino acid sequence alignment of PvFAD3 with the other 34 species of homologous proteins showed that all ω-3 fatty acid dehydrogenases (FAD3) contained three typical conserved histidine-rich structural domains, namely HDCGHG (Hisbox I), PYXGWRISHRTHH (Hisbox II), and HHXIGTHVIHH (Hisbox III) (Figure 2), located at amino acids 95–99, 123–135 and 298–302, respectively (Figure 1A). These three histidine-conserved structural domains had a typical amino acid sequence of fatty acid dehydrogenases on the plasma membrane, which mainly formed the active center of the enzyme and bonded with the 2-valent iron ion to participate in the catalytic action of the enzyme [27]. Of the three histidine-conserved structural domains, the conserved motif of Hisbox I was identical in 35 plant species, and the Hisbox II was “PYHGWRISHRTHH” in 24 plants, whereas “PYNGWRISHRTHH” in six species of *Rosaceae* and two species of *Humulus*, and “PYHGWRISHKTHH” in two species of Flax and Cassia. The conserved motif for Hisbox III was “HHDIGTHVIHH” in 34 species, except for *Durio zibethinus* (“HHNIGTHVIHH”).

The C-terminus of the peptide chain of endoplasmic reticulum retention proteins contain a specific amino acid sequence, the sequence in the membrane proteins is “Lys-Lys-X-X” (KKXX) motif, and “Lys-Asp-Glu-Leu” (KDEL) motif are in the soluble proteins [28]. According to the results of the alignment analysis of amino acid sequence (Figure 2), the FAD3 amino acid sequences of 35 plant species differed considerably at the C-terminus, with only *P. volubilis* and *R. communis* having a consistent and specific endoplasmic reticulum retention signal “KKLA” at the C-terminus. In addition, the other 18 sequences of FAD3 all had the KXKX motif at the C-terminus. Also, 35 FAD3 amino acid sequences were rich in aromatic amino acids such as phenylalanine (Phe, Y) and Tyroxine (Tyr, Y) at the C-terminus.

**Figure 3:**
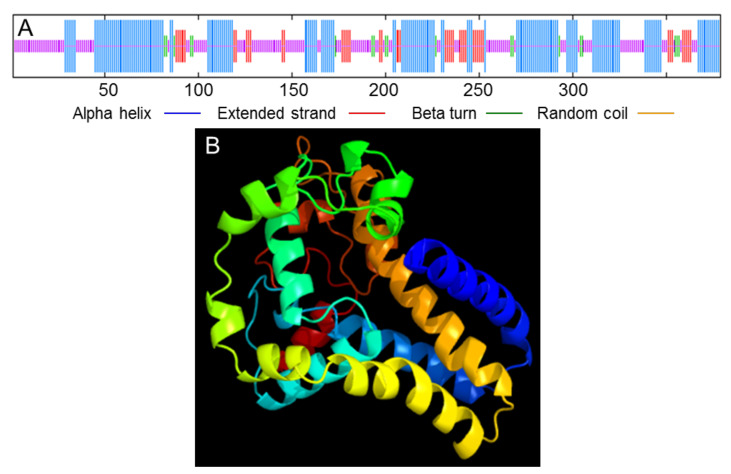
(**A**) Prediction of the secondary structure of PvFAD3 protein. Blue represents alpha helix, red represents extended strand, green represents beta-turn, and yellow represents random coil. (**B**) Tertiary structure models of submits of PvFAD3 protein based on the model of template c4zyoA.

The prediction results of the secondary structure of the PvFAD3 amino acid sequence (Figure 3A) showed that the PvFAD3 contained a large number of α-helices (43.27%) and random coil (39.05%), which may be related to the transmembrane properties of the PvFAD3. Additionally, the extended strand was 12.66%, and the β-sheet was 5.01%. The tertiary structure of the PvFAD3 protein was predicted by homology modeling (Figure 3B), and 250 residues (66% of amino acid sequence) have been modeled with 99.9% confidence by the single highest scoring template.

**Figure 4:**
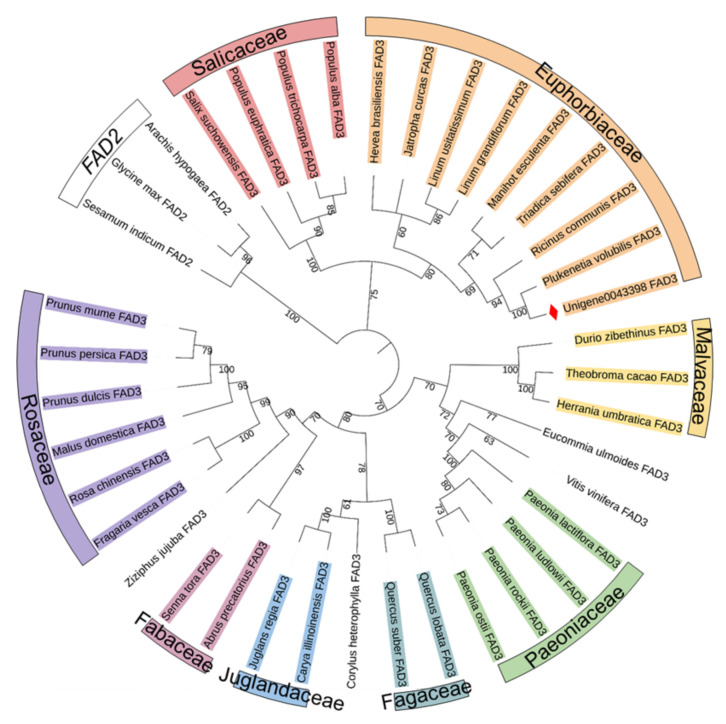
Phylogenetic tree of PvFAD3 and FAD3 from other plants. The phylogenetic tree was constructed by PhyML software with the ML method (1000 bootstrap). The red diamond-shape indicates the position of PvFAD3.

The amino acid sequences of PvFAD3 and the other 35 ω-3 fatty acid dehydrogenases (FAD3) from other plant species were used to build a phylogenetic tree using the maximum likelihood method, with three ω-6 fatty acid dehydrogenases (FAD2) from *A. hypogaea*, *Sesamum indicum*, and *G. max* serving as outgroups (Figure 4). The amino acid sequence of PvFAD3, which was cloned in this study, was the closest to the evolutionary relatives of six species from the Euphorbiaceae family and was clustered into a small group. The FAD3 amino acid sequences of four Salicaceae species were more homologous and clustered into a small group. The amino acid sequences from three species of Malvaceae and four species of Paeoniaceae clustered into one large group with *Eucommia ulmoides* and *Vitis vinifera*, of which three species of Malvaceae had closer evolutionary affinities and clustered into a small group. The amino acid sequences of four species of Paeoniaceae were more homologous. The other 14 plants with higher homology of FAD3 amino acids clustered into one group, with plants in the same family and genus having a higher relationship for the FAD3 protein. The two plants in Fabaceae clustered into a small group, two plants in Fagaceae into a small group, and two plants in Juglandaceae into a small group. Six Rosaceae species had higher homology for the FAD3 amino acid sequences. To conclude, the FAD3 amino acid sequences differed among species, and the evolutionary relationship of the FAD3 proteins is higher between species from the same family.

### 3.2. Expression Pattern of PvFAD3 in Different Tissues 

**Figure 5:**
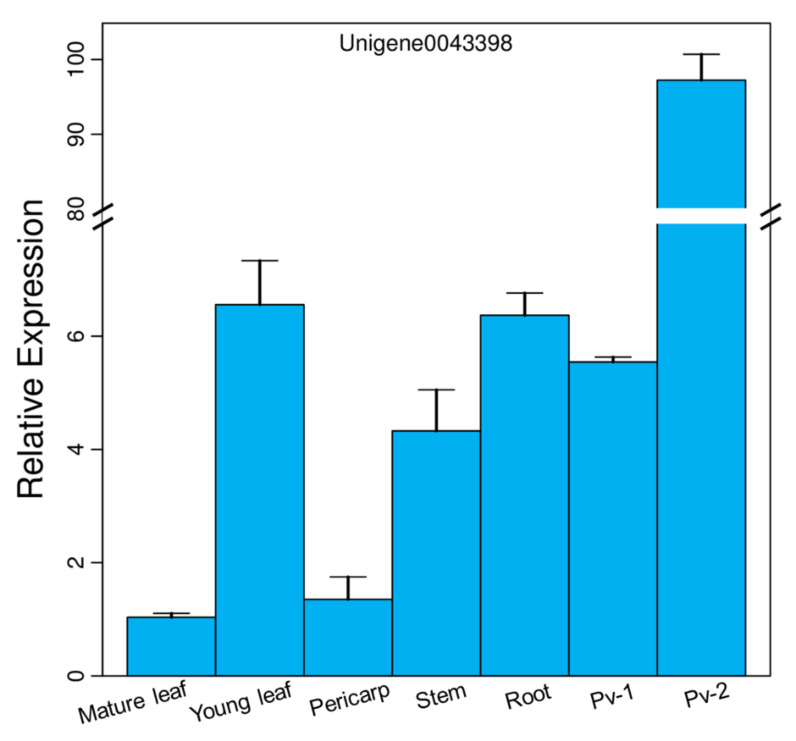
The expression patterns of *PvFAD3*. Error bars represent standard error derived from three replicates. The same letters in the figure represent no significant differences, while different letters indicate significant differences. The data were subjected to Student’s *t*-test.

The expression pattern of the *PvFAD3* in different tissues of *P. volubilis* was analyzed by qRT-PCR (Figure 5), and the relative expression was calculated using the reference gene (Unigene0042747), which showed that *PvFAD3* was expressed in seven different tissues. It was highly expressed during the fast oil accumulation stage of seed development, and its relative expression was 14.83- and 15.27-times higher than that in the initial stage of seed development, and 14.83- and 15.27-times higher than that in young leaves and roots. The *PvFAD3* gene was least expressed in the mature leaves of *P. volubilis*, followed by a lower expression in the pericarp. The expression analysis of photosynthetic and nonphotosynthetic tissues indicated that *PvFAD3* expression was significantly higher in nonphotosynthetic tissues (roots) than in photosynthetic tissues (mature leaves and pericarp) (*p* < 0.05). Compared with the roots, young leaves with vigorous photosynthesis exhibited no difference in the relative expression of the *PvFAD3* gene.

### 3.3. Subcellular Localization of PvFAD3

**Figure 6:**
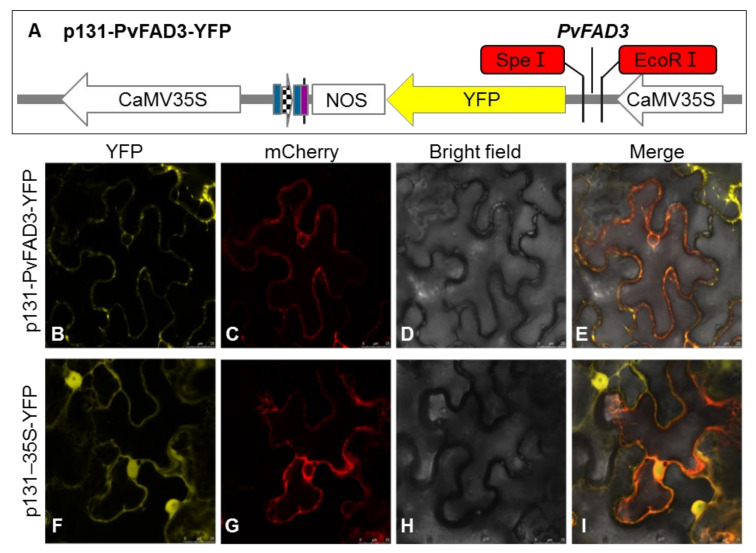
(**A**) Plant fusion vector construction of p131-PvFAD3-YFP; (**B–I**) Subcellular localization analysis of PvFAD3 fusion protein in leaf epidermal cells of *N. benthamiana*; (**B**,**E**) YFP field; (**C**,**G**) red field; (**D**,**H**) bright field; (**E**,**I**) merged pictures. p131-PvFAD3-YFP indicates the fusion protein with PvFAD3, and p131–35S-YFP indicates the empty vector.

To determine the subcellular localization of PvFAD3 protein, the *PvFAD3* gene was cloned into the EcoRI and SpeI sites of the p131-YFP vector by homologous cloning to generate a p131-PvFAD3-YFP fusion vector (Figure 6A). The fusion vector was introduced into *A. tumefaciens* GV3101, the positive clone was picked, and PCR was performed using primers containing the enzyme cut site to obtain a band of the same size as the fragment of PvFAD3, which was sequenced. The p131-PvFAD3-YFP recombinant plasmid with the correct sequencing result was selected and introduced into the lower epidermis of tobacco leaves by injection. After three days of incubation, the transient expression of the *PvFAD3* gene was observed in the epidermal cells of the leaves of tobacco using Leica TCS SP8.

The confocal microscopic analysis showed that the yellow fluorescence from *N. benthamiana* leaves infiltrated with *Agrobacterium* containing p131-PvFAD3-YFP fusion vector was observed mainly in the endoplasmic reticulum when using an ER-rk CD3-959 as an ER marker [25] (Figure 6B), whereas the free YFP signal from *N. benthamiana* leaves infiltrated with *Agrobacterium* containing p131–35S-YFP as the control was diffusely distributed throughout the plasma membranes and organelles (Figure 6F). At the excitation wavelength of 587 nm, the red fluorescence of plasma membrane localization protein (mCherry) was normal (Figure 6C,G). After fusing the YFP, mCherry, and Bright fields, the yellow fluorescence of p131-PvFAD3-YFP fusion vector and red fluorescence of mCherry overlapped and turned orange (Figure 6E). Thus, the PvFAD3 protein was mainly located in the endoplasmic reticulum in cells. 

### 3.4. Fatty Acid Analysis of PvFAD3 Gene-Transformed Tobacco Seeds

**Figure 7:**
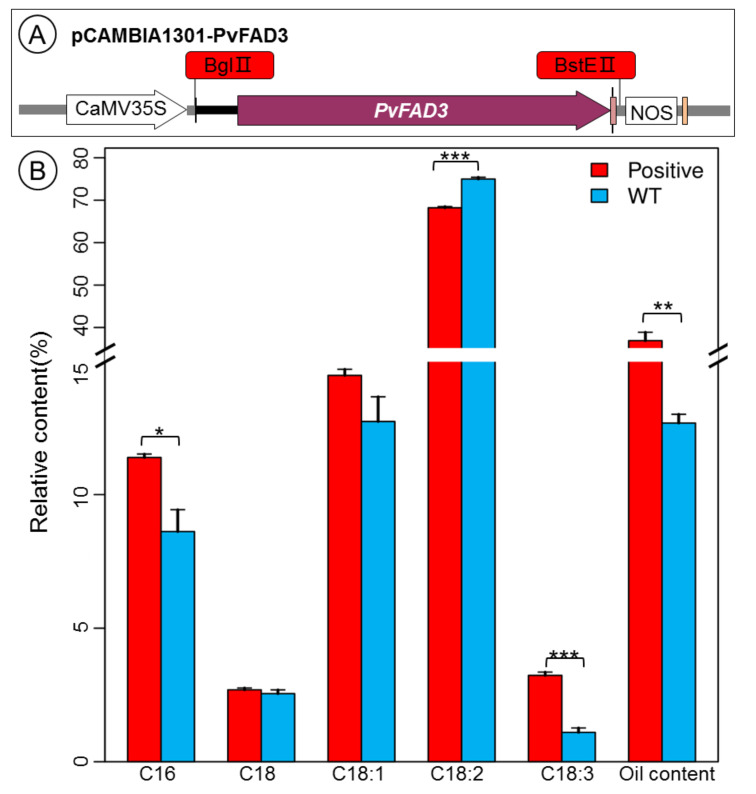
(**A**) The construction of overexpression vector; (**B**) The main fatty acid content analysis of positive transgenic and wild-type tobacco seeds. Positive represents positive transgenic tobacco seeds, WT represents wild-type tobacco seeds. The difference significance was analyzed by ANOVA. * represents significant difference at *p* < 0.05; ** represents significant difference at *p* < 0.01, and *** represent significant difference at *p* < 0.001.

To understand the physiological functions of the *PvFAD3* gene, the ORF fragment of the gene was amplified using the constructed p131-PvFAD3-YFP plasmid as a template, and the PCR product was cloned into the BglII and BstEII restriction enzyme cutting sites of the pCAMBIA1301 vector by homologous recombination (Figure 7A). After transformation using *A. tumefaciens*, positive clones were selected and analyzed by PCR using primers containing the restriction enzyme cutting site to obtain bands consistent with the fragment size of PvFAD3 (Supplementary Figures S1 and S2). The recombinant pCAMBIA1301-PvFAD3 plasmid was sequenced to confirm its sequence and then used to obtain transgenic tobacco seedlings overexpressing the *PvFAD3* gene via *A. tumefaciens*-mediated genetic transformation by the tobacco leaf disc method (Supplementary Figure S3A–D). Wild-type and positive transgenic tobacco leaves were used as materials for PCR using specific primers (35S-F/FAD3-R2(562)), respectively. The results showed that 13 transgenic tobacco seedlings numbered 2–9, 11–14, and 16 contained the *PvFAD3* gene (Supplementary Figure S3E).

Mature tobacco seeds were harvested from wild-type, 13 positive transgenic tobaccos were grown in a greenhouse under normal light and water, and the fatty acid composition in mature seeds of wild-type and positive transgenic tobacco was analyzed using gas chromatography (Figure 7B). Compared with the wild type, the fatty acid composition was changed greatly in the transgenic tobacco seeds. In particular, the content of alpha-linolenic acid was remarkably higher in positive transgenic tobacco seeds than in wild-type (*p* < 0.001). Simultaneously, oil content (*p* < 0.01) and palmitic acid content (*p* < 0.05) were also increased significantly in positive transgenic tobacco seeds compared with wild-type, separately. Conversely, linoleic acid content was significantly lower in positive transgenic tobacco seeds than in wild-type (*p* < 0.001). These results show that the overexpression of the *PvFAD3* in tobacco could significantly enhance the conversion of linoleic acid to α-linolenic acid and increase the oil content of the transgenic tobacco seeds.

## 4. Discussion

α-Linolenic acid is an essential ω-3 fatty acid for human growth and development, as it promotes brain development, prevents neurological and cardiovascular diseases, lowers blood lipids, and slows the aging process [29]. In daily life, α-linolenic acid is relatively low in traditional diets such as peanuts, soybeans, maize, oil tea, and olives [30], and the balance of fatty acid fractions concerns genetic improvement of oilseed plants. 

With the continuous development of oil plants, special oil plants rich in α-linolenic acid such as *Perilla frutescens* [31], *Salvia hispanica* [32], *Linum usitatissimum* [33], *P. volubilis* [34], and *P. suffruticosa* [35] seeds have been discovered as new sources of α-linolenic acid and dietary supplements. The high yield and oil content of *P. volubilis* seeds, rich in both α-linolenic acid and linoleic acid, exhibit greater advantages than those of other oil plants and have greater potential for exploitation. The cloning, expression, and functional analysis of PvFAD3, a key enzyme for α-linolenic acid biosynthesis in *P. volubilis*, are therefore of great theoretical value.

In this study, the *PvFAD3* gene was obtained by PCR homologous cloning based on the transcriptome data of *P. volubilis* in our previous studies [21]. The bioinformatics analysis of PvFAD3 showed that the full-length DNA sequence of PvFAD3 was 1871 bp, including eight exons and seven introns, encoding 379 amino acids, with a molecular weight of 43.53 kDa and an isoelectric point of 7.79. The PvFAD3 cloned in this study was similar to that of *R. communis*, *J. curcas*, and *Hevea brasiliensis*, the members of Euphorbiaceae. The closer genetic distance of ω-3 fatty acid dehydrogenase (FAD3) in the same family in the phylogenetic analysis indicated that the FAD3 was more conserved in the biological structure of species within the same family and with higher homology.

The structural analysis of the amino acid sequence of PvFAD3 protein showed that the C-terminus contained a distinct endoplasmic reticulum retention signal (KKLA motif), but there was no signal peptide or restriction enzyme cutting site. This finding supports the conclusion that the PvFAD3 protein is primarily localized to the endoplasmic reticulum via subcellular localization, as observed in tobacco leaf epidermis. McCartney et al. [36] reported that the C-terminal sequences rich in aromatic amino acids can localize fatty acid dehydrogenase to the endoplasmic reticulum membrane, and the C-terminal amino acid sequences of the ω-3 fatty acid dehydrogenase (FAD3) of the 35 plants were rich in aromatic amino acids (Figure 2). In addition, the amino acid sequence of the PvFAD3 protein contained the “KKLA” sequence. To sum up, the PvFAD3 protein was a typical ω-3 fatty acid dehydrogenase protein in the endoplasmic reticulum, that may bind tightly to the substrate and have a high catalytic efficiency, facilitating the conversion of linoleic acid to α-linolenic acid. Based on the structural comparison of the amino acid sequences of the different species, ω-3 fatty acid dehydrogenase of all 35 plant species had 3 specific histidine-conserved regions (Hisbox I, Hisbox II, and Hisbox III) that formed covalent bonds with amino acids embedded in the endoplasmic reticulum membrane and thus anchored to the endoplasmic reticulum. Only a few amino acid residues of plants in the conserved regions of Hisbox II and Hisbox III were changed. Predictably, the histidine-conserved regions in the ω-3 fatty acid dehydrogenase were conserved and stable in higher plants; this finding is in accordance with the results of the analysis of the histidine-conserved regions of ω-6 and ω-3 fatty acid dehydrogenases by Guan et al. (2013) [28]. In the present study, the positions of the three histidine-conserved regions were annotated on the full-length sequence structure of the PvFAD3 (Figure 1), and all three histidine-conserved regions were located in the intron regions, with Hisbox I and Hisbox II in the first intron region and Hisbox III in the fourth intron region. This observation further confirmed that introns play a crucial role in ensuring the conserved function of the gene during the evolution of species [37].

The expression patterns of genes can provide direction for their functional roles. Several studies have confirmed that the *FAD3* is highly expressed in nonphotosynthetic tissues such as roots, flowers, and seeds of nonoil plants, including *A. thaliana* [38], *Gossypium hirsutum* [39], and *Oryza sativa* [40]. In contrast, it was specifically highly expressed in the seeds of oil plants, such as *P. frutescens* [41], *G.* *max* [42], *L. usitatissimum* [14], and *A. hypogaea* [15], which are closely related to the biosynthesis of α-linolenic acid in seeds. In the present study, the expression patterns of *PvFAD3* in different tissues such as roots, stems, leaves, seeds, and pericarp showed that *PvFAD3* was expressed in different parts and was specifically upregulated in the rapid accumulation stage of seed oil. In contrast, the relative expression in the nonphotosynthetic roots was not significantly different from that in photosynthetic new leaves. Thus, the expression of *PvFAD3* may not be directly related to photosynthesis in different tissues.

*FAD3* is a key gene for α-linolenic acid biosynthesis in plants, which catalyzes the formation of α-linolenic acid from linoleic acid by introducing a third double bond between C15 and C16 of linoleic acid [43]. The study of expression patterns of the FAD3 in different oilseed plants showed that the high content of ALA in plant seeds might have a stronger correlation with the expression activity of the *FAD3*. To explore the possible molecular mechanism by which *PvFAD3* positively regulates the biosynthesis of α-linolenic acid, we investigated the regulating effects of *PvFAD3* on the expression levels of two stages of developing seeds (the initial stage, Pv-1 and the fast oil accumulation stage, Pv-2) in *P. volubilis*. The expression of *PvFAD3* was downregulated at Pv-1 and significantly upregulated at Pv-2, consistent with the dynamic change of α-linolenic acid content at various stages of seed development [21], thus further demonstrating the close correlation between *PvFAD3* expression and α-linolenic acid accumulation. To further validate the biological function of the *PvFAD3*, a vector overexpressing the *PvFAD3* was constructed and overexpressed in tobacco seeds, which significantly increased the content of α-linolenic acid (2.13%), significantly decreased the content of linoleic acid (6.78%), and obviously increased the oil content (24.22%) and palmitic acid (2.77%). The stearic and oleic acid contents did not change significantly. This is consistent with the finding that the overexpression of the FAD3 gene significantly increased α-linolenic acid content in plants such as *P. frutescens* [44], *J. curcas* [14], *G. max* [13], *O. sativa* [40], and *L.* *esculentum* [12].

## 5. Conclusions

In conclusion, the full-length sequence of *PvFAD3* was successfully cloned and analyzed using bioinformatics. The gene was subcellularly localized to the endoplasmic reticulum, and overexpression of the *PvFAD3* gene in tobacco showed a significant increase in α-linolenic acid content and oil content in transgenic plant seeds. Collectively, the *PvFAD3* gene of *P. volubilis* was confirmed as a key enzyme gene for α-linolenic acid synthesis, thus indicating that the *PvFAD3* gene can be used for fatty acid fraction improvement in oilseed plants. The results of this study would help reveal the molecular mechanism of α-linolenic acid biosynthesis in oil plants, provide a basis for further research on its regulatory mechanism, and develop a foundation for high yield and high quality of oil plants.

## Data Availability

Not applicable.

## Supplementary Materials

The following supporting information can be downloaded at: https://www.mdpi.com/article/10.3390/genes13030450/s1, Figure S1: Amplification results of the fusion vector; Figure S2: Amplification results of the overexpression vector; Figure S3 (A–D): The genetic transformation of transgenic tobacco; (E): PCR detection of positive plants of transgenic tobacco; Table S1: Primers used in this research; Table S2: Alignment description of endoplasmic reticulum ω-3 fatty acid desaturases.

## Abbreviations

α-Linolenic acid (C18:3, ALA); Docosahexaenoic acid (DHA, C22:6); Eicosapentaenoic acid (EPA, C20:5); ω-3 fatty acid desaturase (*FAD3*); β-Glucuronidase (GUS); Lys-Asp-Glu-Leu (KDEL); Lys-Lys-X-X (KKXX); kanamycin (Kan); Open reading frames (ORF); Theoretical isoelectric point (pI); phenylalanine (Phe, Y); real-time reverse transcription-polymerase chain reaction (qRT-PCR); Tyroxine (Tyr, Y).
