# Supplementary material for: Overexpression of PvFAD3 Gene from Plukenetia volubilis Promotes the Biosynthesis of α-Linolenic Acid in Transgenic Tobacco Seeds
Source: Genes (Basel). 2022 Feb 28;13(3):450. doi: 10.3390/genes13030450 (PMC8951128; doi:10.3390/genes13030450)
Supplement: Supplementary file 1 [file genes-13-00450-s001.zip › genes-1612732-supplementary.pdf]

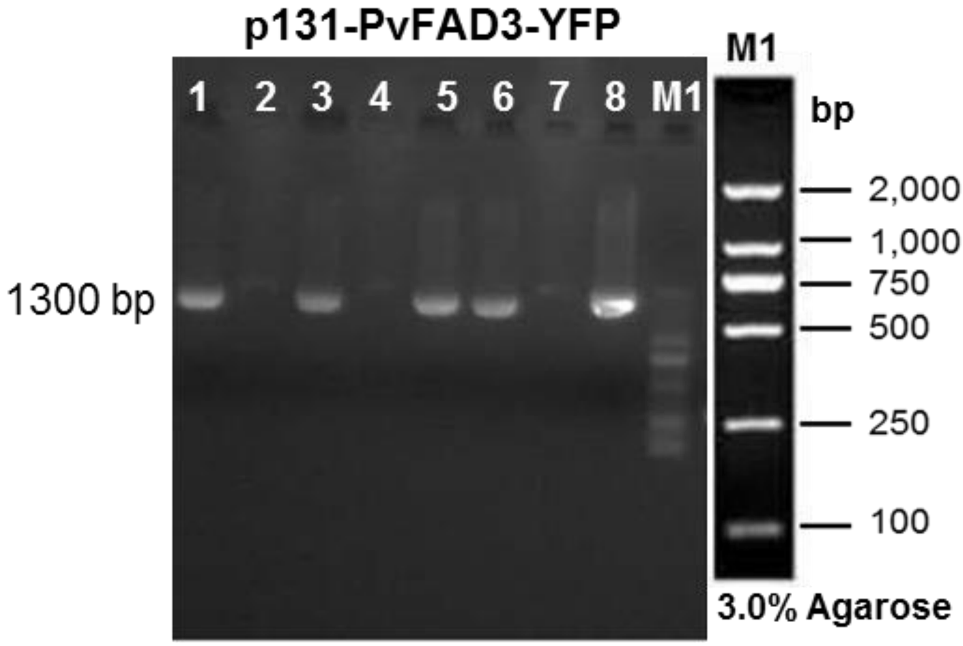

Figure S1. Amplification results of the fusion vector. Number 1, 3, 5, 6 and 8 are positive clones.

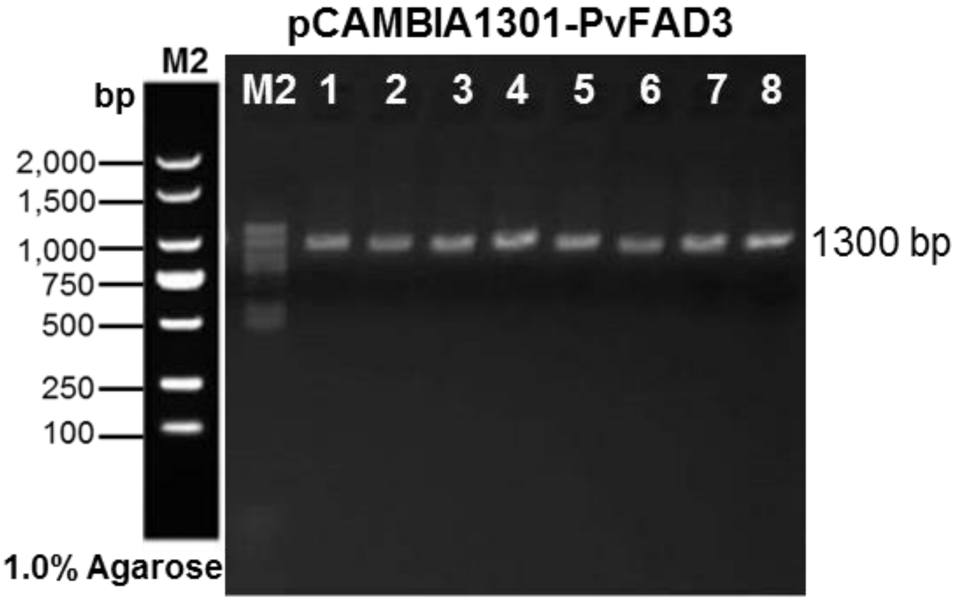

Figure S2. Amplification results of the overexpression vector. Number 1-8 are positive clones.

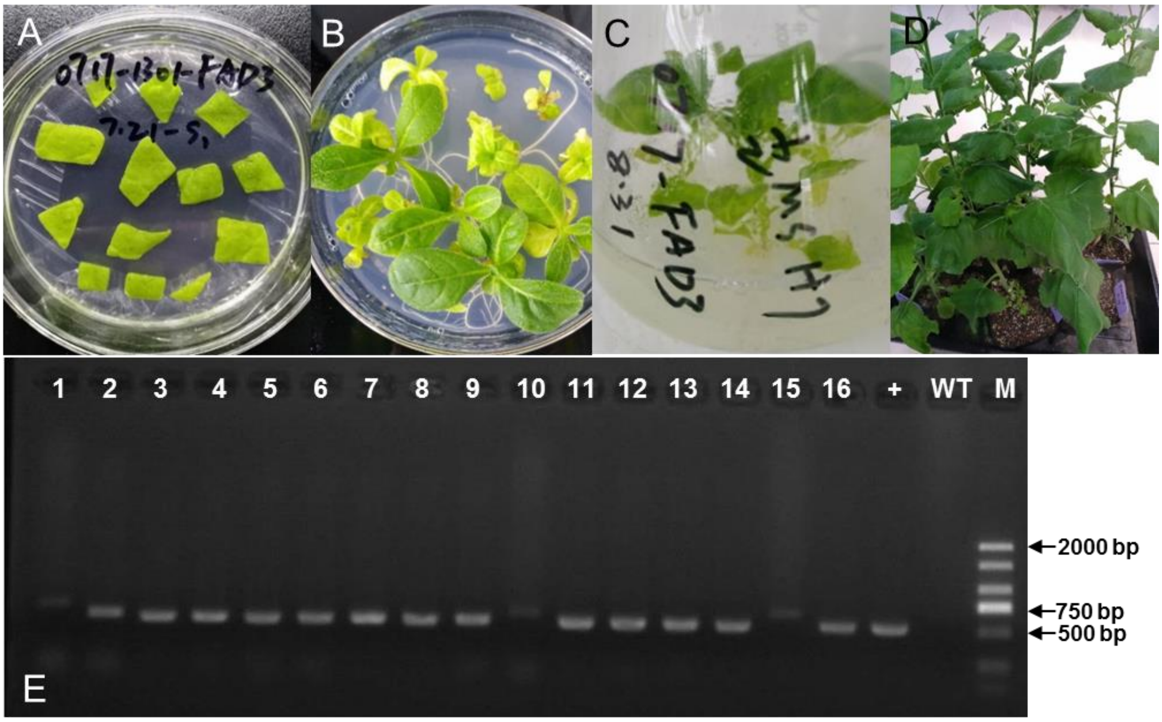

Figure S3. (A-D): The genetic transformation of transgenic tobacco; (E): PCR detection of positive plants of transgenic tobacco. Number 2-9, 11-14 and 16 are positive transgenic tobaccos.

Table S1. Primers used in this research

Primer names	Primer sequence (5'-3')
FAD3-F	ATGCAGACCATGGATATTTC
FAD3-R	TTAAACTAACTTGGTTTTTGC
YFP-FAD3-F	AagagacaggatccgaattcATGCAGACCATGGATATTTC
YFP-FAD3-R	A cctccgaccggtgcactagtAACTAACTTGGTTTTTGC
1301-FAD3-F	gactcttgaccatggtagatctaATGCAGACCATGGATATTTC
1301FAD3-R	gggaaattcgagctggtcaccTTAAACTAACTTGGTTTTTGC
35S-F	GACGCACAATCCCACTATCC
FAD3-R2(562):	CTTTGCCTGGACTCCTTGAC
Unigene0042747-F	CCAGTATGTCGGGGTCTTCC
Unigene0042747-R	CCGTTTCTCTCTCTCGGTCAG
Unigene0043398-F	GCAGCCAAAGCAATGACAACA
Unigene0043398-R	CGGGAATAGACAGGGAGCA

Note: Underline indicate the restriction enzyme cutting site; Unigene0042727 is the reference gene. Unigene0043398 is the transcript of *PvFAD3*.

Table S2. Alignment description of endoplasmic reticulum ω-3 fatty acid desaturases

Description	Scientific Name	Max Score	Total Score	Query Cover	E-value	Per. ident	Acc. Len	Accession
fatty acid desaturase	*Plukenetia volubilis*	782	782	100%	0	100	379	QGR25673.1
omega-3 fatty acid desaturase, endoplasmic reticulum	*Ricinus communis*	680	680	100%	0	87.07	378	XP_015578794.1
PREDICTED: omega-3 fatty acid desaturase, endoplasmic reticulum	*Populus euphratica*	612	612	91%	0	83.91	378	XP_011043784.1
omega-3 desaturase family protein	*Salix suchowensis*	609	609	91%	0	83.33	384	KAG5253823.1
acyl-lipid omega-3 desaturase (cytochrome b5), endoplasmic reticulum-like	*Populus alba*	630	630	96%	0	82.47	384	XP_034893756.1
acyl-lipid omega-3 desaturase (cytochrome b5), endoplasmic reticulum	*Populus trichocarpa*	623	623	96%	0	81.64	383	XP_002298309.1
omega-3 fatty acid desaturase	*Triadica sebifera*	635	635	96%	0	81.37	372	ABM68629.1
omega-3 fatty acid desaturase, endoplasmic reticulum-like	*Manihot esculenta*	625	625	97%	0	81.25	374	XP_021610352.1
acyl-lipid omega-3 desaturase (cytochrome b5), endoplasmic reticulum-like	*Hevea brasiliensis*	642	642	99%	0	80.16	377	XP_021666259.1
omega-3 fatty acid desaturase, endoplasmic reticulum-like	*Jatropha curcas*	615	615	98%	0	79.3	377	NP_001292929.1
omega-3 fatty acid desaturase	*Eucommia ulmoides*	600	600	93%	0	78.25	426	ARQ20744.1
acyl-lipid omega-3 desaturase (cytochrome b5), endoplasmic reticulum-like	*Quercus lobata*	607	607	94%	0	78.21	384	XP_030969358.1
omega-3 fatty acid desaturase	*Paeonia ostii*	600	600	93%	0	77.4	435	AMO44419.1
omega-3 fatty acid desaturase	*Paeonia lactiflora*	596	596	93%	0	77.12	435	AJA36814.1
omega-3 fatty acid desaturase	*Paeonia rockii*	598	598	93%	0	77.12	435	QBA82276.1
PREDICTED: omega-3 fatty acid desaturase, endoplasmic reticulum	*Fragaria vesca subsp. vesca*	596	596	96%	0	76.78	387	XP_004294292.1
omega-3 fatty acid desaturase, endoplasmic reticulum	*Rosa chinensis*	599	599	96%	0	76.23	385	XP_024158047.1
acyl-lipid omega-3 desaturase (cytochrome b5), endoplasmic reticulum-like	*Malus domestica*	590	590	94%	0	76.19	388	XP_008384790.2
omega-3 fatty acid desaturase, endoplasmic reticulum-like	*Senna tora*	598	598	95%	0	76.1	391	KAF7816112.1

Description	Scientific Name	Max Score	Total Score	Query Cover	E-value	Per. ident	Acc. Len	Accession
PREDICTED: acyl-lipid omega-3 desaturase (cytochrome b5), endoplasmic reticulum	*Vitis vinifera*	591	591	96%	0	75.89	386	XP_002277573.1
PREDICTED: acyl-lipid omega-3 desaturase (cytochrome b5), endoplasmic reticulum-like	*Prunus mume*	590	590	94%	0	75.63	384	XP_008220937.1
acyl-lipid omega-3 desaturase (cytochrome b5), endoplasmic reticulum	*Prunus persica*	591	591	94%	0	75.63	384	XP_007205342.1
fatty acid desaturase 3	*Corylus heterophylla*	595	595	97%	0	75.47	386	AEF80000.1
acyl-lipid omega-3 desaturase (cytochrome b5), endoplasmic reticulum-like	*Prunus dulcis*	590	590	94%	0	75.35	384	XP_034219955.1
acyl-lipid omega-3 desaturase (cytochrome b5), endoplasmic reticulum-like	*Quercus suber*	609	609	98%	0	75.34	387	XP_023919451.1
fatty acid desaturase 3	*Linum usitatissimum*	600	600	97%	0	75.13	391	AFJ53089.1
omega-3 fatty acid desaturase, endoplasmic reticulum-like	*Carya illinoinensis*	605	605	99%	0	74.74	379	XP_042954309.1
omega-3 fatty acid desaturase	*Paeonia ludlowii*	597	597	96%	0	74.66	438	QBQ82059.1
omega-3 fatty acid desaturase	*Linum grandiflorum*	608	608	97%	0	74.6	393	BAG70950.1
acyl-lipid omega-3 desaturase (cytochrome b5), endoplasmic reticulum	*Herrania umbratica*	606	606	99%	0	74.54	391	XP_021279437.1
omega-3 fatty acid desaturase, endoplasmic reticulum-like	*Juglans regia*	606	606	100%	0	74.28	380	XP_018835457.1
omega-3 fatty acid desaturase, endoplasmic reticulum-like	*Durio zibethinus*	602	602	98%	0	74.13	388	XP_022756628.1
omega-3 fatty acid desaturase, endoplasmic reticulum-like	*Ziziphus jujuba*	592	592	98%	0	74.06	377	XP_015873733.1
PREDICTED: acyl-lipid omega-3 desaturase (cytochrome b5), endoplasmic reticulum isoform X1	*Theobroma cacao*	600	600	99%	0	74.01	391	XP_007016081.2
omega-3 fatty acid desaturase, endoplasmic reticulum-like	*Abrus precatorius*	590	590	97%	0	73.58	382	XP_027334605.1
